# The Smoke Nuisance

**Published:** 1882-09

**Authors:** 


### THE SMOKE NUISANCE.

" Regarding the importance of the gas-supply as it exists at present, we find from a Government return that the capital invested in gas works in England, other than those of local authorities, amounts to £30,000,000 : in these 4,281,048 tons of coal are converted annually, producing 43,000 million cubic feet of gas, and about 2,800-000 tons of coke ; whereas the total amount of coal annually converted in the United Kingdom may be estimated at 9,000,000 tons, and the by-products therefrom at 500,000 tons of tar, 1,000,000 tons of ammonia liquor, and 4,000,000 tons of coke, according to the returns kindly furnished me by the managers of many of the gas works and corporations. To these may be added, say, 120,000 tons of sulphur, which up to the present time is a waste product. Taking the coal used, 9,000,000 tons, at 12s., equal £5,400,000, the by-products at gas works exceed in value the coal used by nearly £3,000,000. In using raw coal for heating purposes, these valuable products are not only absolutely lost to us, but in their stead we are favored with those semi-gaseous by-products in the atmosphere too well known to the denizens of London and other large towns as smoke. Professor Robert has calculated that the soot in a pall hanging over London on a winter's day amounts to fifty tons; and that the carbonic oxide, a poisonous compound, resulting from the imperfect combustion of coal, may be taken as at least five times that amount. The fine dust resulting from the imperfect combustion of coal is mainly instrumental in the formation of fog; each particle of solid matter attracting to itself aqueous vapor; these globules of fog are rendered particularly tenacious and disagreeable by the presence of tar-vapor, another result of imperfect combustion of raw fuel, which might be turned to much better account at the dye-works. The most effectual remedy would result from a general recognition of the fact that, wherever smoke is produced fuel is being consumed wastefully, and that all our caloric effects, from the largest down to the domestic fire, can be realized as completely and more economically, without allowing any of the fuel employed to reach the atmosphere unburnt. This most desirable result may be effected by the use of gas for all heating purposes with or without the addition of coke or anthracite.—*Dr. Siemens in British Medical Journal.*

